# Compound danshen tablet ameliorated aβ_25-35_-induced spatial memory impairment in mice via rescuing imbalance between cytokines and neurotrophins

**DOI:** 10.1186/1472-6882-14-23

**Published:** 2014-01-14

**Authors:** Yan Teng, Meng-Qi Zhang, Wen Wang, Li-Tao Liu, Li-Ming Zhou, Shi-Kun Miao, Li-Hong Wan

**Affiliations:** 1 Department of Pharmacology, West China School of Preclinical and Forensic Medicine, Sichuan University, Chengdu, Sichuan 610041, PR China; 2 Basic Medicine 2009 undergraduate students, West China School of Preclinical and Forensic Medicine, Sichuan University, Chengdu, Sichuan 610041, PR China; 3 Sichuan University “985 project -- Science and technology innovation platform for novel drug development”, Sichuan University, Chengdu, Sichuan 610041, PR China

**Keywords:** Compound danshen tablet, Spatial memory impairment, ChAT, RACK1, BDNF

## Abstract

**Background:**

Compound Danshen Tablet (CDT), a Traditional Chinese Medicine, has recently been reported to improve spatial cognition in a rat model of Alzheimer’s disease. However, in vivo neuroprotective mechanism of the CDT in models of spatial memory impairment is not yet evaluated. The present study is aimed to elucidate the cellular mechanism of CDT on Aβ_25-35_-induced cognitive impairment in mice.

**Methods:**

Mice were randomly divided into 5 groups: the control group (sham operated), the Aβ_25-35_ treated group, the positive drug group, and large and small dosage of the CDT groups, respectively. CDT was administered at a dose of 0.81 g/kg and 0.405 g/kg for 3 weeks. The mice in the positive drug group were treated with 0.4 mg/kg of Huperzine A, whereas the mice of the control and Aβ_25-35_ treated groups were administrated orally with equivalent saline. After 7 days of preventive treatment, mice were subjected to lateral ventricle injection of Aβ_25-35_ to establish the mice model of Alzheimer’s disease. Spatial memory impairment was evaluated by Morris water maze test. Choline acetyltransferase (ChAT) contents in hippocampus and cortex were quantified by ELISA. The levels of cytokines, receptor of activated protein kinase C1 (RACK1) and brain-derived neurotrophic factor (BDNF) in hippocampus were measured by RT-PCR and ELISA.

**Results:**

The results showed that Aβ_25-35_ caused spatial memory impairment as demonstrated by performance in the Morris water maze test. CDT was able to confer a significant improvement in spatial memory, and protect mice from Aβ_25-35_-induced neurotoxicity. Additionally, CDT also inhibited the increase of TNF-α and IL-6 level, and increased the expression of choline acetyltransferase (ChAT), receptor of activated protein kinase C1 (RACK1) and brain-derived neurotrophic factor (BDNF) in brain as compared to model mice.

**Conclusion:**

These findings strongly implicate that CDT may be a useful treatment against learning and memory deficits in mice by rescuing imbalance between cytokines and neurotrophins.

## Background

Alzheimer’s disease (AD) is an age-related progressive neurodegenerative disorder associated with impairment of learning and memory function partly caused by loss of cholinergic neuron in hippocampus and cortex [1]. Deposits of fibrillar amyloid-β (Aβ) protein and neurofibrillary tangles (NFTs) in the brain are two pathological hallmarks of Alzheimer’s disease [2]. Currently, activation of inflammatory mediators [3-5] and reduction of BDNF [6] have been proposed as the potential mechanism for Alzheimer’s disease. Although growing evidences have reported that anti-inflammation [7] and up-regulation of BDNF [8] are effective on the memory impairment and neural damage. Up to now, there is still no drugs have been developed to retard the pathologic processes of AD, and the pharmacotherapy focuses mainly on relieving cognitive symptoms using cholinesterase inhibitors [9] and NMDA receptor antagonist [10].

In recent years, many traditional herbal formulations have also been reported to significantly improve cognitive function in clinical trials [11,12] and preclinical experiments [13], such as Compound Danshen Tablets (CDT) [14]. Compound Danshen Tablet consisted of *Savia miltiorrhiza*, *Panax Notoginseng* and *Borneol*. Multiple main active ingredient of Compound Danshen Tablets (CDT) has been demonstrated to possess therapeutic effects against Alzheimer’s disease in animal model [15,16]. However, to the best of our knowledge, there is no available study that evaluates the effects of Compound Danshen Tablets (CDT) on neuroinflammation and neurotrophin levels in an experimental mice model of Alzheimer’s disease.

Huperzine A is an active natural compound isolated from *Huperzia serrata (Thunb) Trev*. Due to its potent, selective inhibitory effect of acetylcholinesterase, Huperzine A was first approved by State Food and Drug Administration of China for the treatment of Alzheimer’s disease (AD) in 1994 [17]. In this study, we chose it as the positive drug to assess the effect of CDT.

Hence, to determine if CDT attenuated Aβ_25-35_-induced neuroinflammation and restoring the neurotrophin level in mice, we established the AD model mice model by i.c.v. Aβ_25-35_ to evaluate the potential effect and mechanism of CDT on the cognitive function impairment. Our results first demonstrated that CDT largely restored the cognitive function and neural damage in Aβ_25-35_-induced mice via up-regulating the expressions of choline acetyltransferase (ChAT), Receptors for activated C kinase1 (RACK1) and brain derived neurotrophic factor (BDNF) and down-regulating the levels of IL-6 and TNF-α.

## Methods

### Animals

Male Kunming mice, 8 ~ 10 weeks old, weighing 38 ~ 42 g, were obtained from the Experimental Animal Center at Sichuan University. The mice were housed in plastic cages with hardwood chip bedding in an air-conditioned room at 23 ± 2°C and 55 ± 5% humidity, and with a 12 h light/dark cycle on basal diet (animal center). The animal handlings and experimental procedures were approved by the Committee on the Ethics of Animal Experiments of Sichuan University (Permit Number: 2003–149).

### Drugs and reagents

Compound Danshen Tablet (CDT) was produced by Hutchison Whampoa Guangzhou Baiyunshan Chinese Medicine Co, Ltd (batch number: Z44023372, Guangzhou, China). The formula consisted of *Savia miltiorrhiza*, *Panax Notoginseng*, *Borneol* in proportions of 450:141:8. The main active ingredient were identified as tanshinone, cryptotanshinone, dihydrotanshinone I, tanshinone IIA, salvianolic acid B, sodium danshensu, rosmarinic acid and lithospermic acid by HPLC fingerprint [18]. The tablets were grinded and dissolved in sterile saline before use.

Huperzine A (Purity ≥ 99%, HPLC), acetylcholinesterase inhibitor, was produced by Chengdu Must Biotechnology Co, Ltd (batch number: MusT-11041101, Chengdu, China) and also dissolved in sterile saline before use. Coomassie brilliant blue protein assay kit was purchased from Nanjing Jiancheng Bioengineering Institute (Nanjing, China). Amyloid β-protein fragment 25–35 (Aβ_25-35_) was purchased from Beijing Biosynthesis Biotechnology Co, LTD (Beijing, China).

### Experimental instruments

The MT-200 Morris water maze system (Chengdu TME Technology Co, Ltd, Chengdu, China); ZS-3 Microplate Reader (Beijing Xinfeng Electromechanical Technology Co, Ltd, Beijing, China).

### Aged Aβ_25-35_ peptide preparation

The Aβ_25-35_ was dissolved in sterile saline at a concentration of 2 g/L and incubated at 37°C for 7 days to allow for fibril formation as described previously [19].

### Mice screening and allocation

MT-200 Morris water maze is a large round pool (100 cm in diameter, 50 cm in height) filled with water at 12 cm depth and 25 ± 2°C. Water was made opaque with nontoxic white colored dye. The pool was divided arbitrarily into four equal quadrants. A platform was in the pool placed 2 cm below the water surface in the middle of one quadrant. The position of the platform was unchanged in the whole process. The mouse was allowed to swim until it finds the hidden platform. Twice training trails per day were conducted for four consecutive days, with an inter-trial interval of 15 min. On day 5, all the mice found the platform within 60 s were randomly divided into 5 groups (n = 10): A-the control group (sham operated), B-the Aβ_25-35_ treated group, C-the positive drug group (Huperzine A), D-large dose of Compound Danshen Tablets group (LCDT) and E-small dose of Compound Danshen Tablets (SCDT) group. The mice in the large (LCDT), and small dose groups (SCDT) were administrated orally with doses of 0.81 g/kg and 0.405 g/kg (equivalent to 1 and 0.5 times of an adult human dosage), respectively for 7 consecutive days. The mice in the positive drug group were treated with 0.4 mg/kg of Huperzine A, whereas the mice of the control and Aβ_25-35_ treated groups were administrated orally with equivalent saline.

### Experimental procedure

After 7 days of preventive treatment, mice were lightly anaesthetized with ether and subjected to lateral ventricle injection of vehicle (saline) or Aβ_25-35_ (2 mg/mL) 5 μl. The needle was left in place for 10s following the injection. Then, the mice received orally either saline (control and Aβ_25-35_ treated group) or 0.4 mg/kg Huperzine A and 0.81 and 0.405 g/kg Compound Danshen Tablets for consecutive 14 days.

### Behavioral assessment

Spatial learning and memory abilities of mice were assessed in the Morris water maze, including place navigation trial and spatial probe trial. In place navigation trial, the escape latency was recorded for 60 s for four trials daily on day 19 to 21. On day 22, a spatial probe trial was conducted by removing the platform and placing the mouse next to and facing the pool wall and each mouse was allowed to swim freely for 60 s. During the probe trial, the number of platform crossings and the time spent in the goal quadrant were recorded.

### Brain tissue preparation

After the probe trial, all mice were sacrificed by decapitation. The brain tissues were removed immediately. One hemi-brain was fixed in 4% paraformaldehyde for histopathology assay. The hippocampus of other hemi-brain was snap frozen in liquid nitrogen and stored at −80°C for use in ELISA and RT-PCR analysis.

### Brain histopathology

Coronal sections (4 μm) through the brain were embedded in paraffin and stained with hematoxylin and eosin (H&E) for microscopic evaluation of neuronal damage. The damage induced by Aβ_25-35_ was evaluated as follows: three sections from each hippocampus being used for the morphometric analysis. Light-microscope images were photographed, and the total pyramidal cell numbers per millimeter in the hippocampus and in the subregions CA3 were measured on the photographs and then averaged to give a single value. Data were calculated as average of six slices for each group.

### Quantification of Aβ_1-42_, ChAT, IL-6, TNF-α, BDNF and RACK1 by ELISA

The Aβ_1-42_, ChAT, IL-6, TNF-α, BDNF and RACK1 concentration in hippocampus (n = 5) were measured using commercial available ELISA kits (R&D, USA), according to the manufacturer’s protocol. Duplicate samples were analyzed for each data point. The amount of Aβ_1-42_, ChAT, IL-6, TNF-α, BDNF and RACK1 were determined by absorbance in 450 nm respectively.

### RT-PCR

Hippocampus from 5 animals in each group was used for RT-PCR. The tissue was rapidly removed, dissected and stored at −80°C. Total RNA (1 μg), obtained using RNAprep pure tissue kit according to the manufacturer’s instructions, was subjected to reverse transcription with an oligo-dT18 primer, recombinant RNAsin, and M-MLV reverse transcriptase (all Fermentas, EU). PCR was performed with a gene amp PCR system thermal cycler (Eppendorf, German), and Taq DNA polymerase (Fermentas). The primers sequences and conditions used in this study were listed in Table 1.

**Table 1:** Primer sequence and condition for RT-PCR

**Gene**	**Forward primer (5′ to 3′)**	**Reverse primer (5′ to 3′)**	**Condition**	**PCR product length (bp)**
β-actin	TGGAATCCTGTGGCATCCA	TAACAGTCCGCCTAGAAGCA	95°C 3 min, (94°C 45 s, 55°C 45 s, 72°C 45 s) for 32 cycles, 72°C 10 min	343
IL-6	GAGGATACCACTCCCAACAGACC	AAGTGCATCATCGTTGTTCATACA	95°C 5 min, (94°C 30 s, 58°C 30 s, 72°C 60 s) for 35 cycles, 72°C 10 min	141
TNF-α	GGCAGGTCTACTTTGGAGTCATTGC	ACATTCGAGGCTCCAGTGAATTCGG	95°C 5 min, (94°C 30 s, 58°C 30 s, 72°C 60 s) for 35 cycles, 72°C 10 min	300
RACK1	ACCAACAAGGCGATTTGTCG	GCAGACACCCAGAGTATTCCATA	94°C 2 min, (94°C 30 s, 52°C 30 s, 72°C 2 min) for 32 cycles, 72°C 8 min	136
BDNF	AGCCTCCTCTGCTCTTTCTGCTGGA	CTTTTGTGTATGCCCCTGCAGCCTT	95°C 5 min, (95°C 45 s, 58°C 45 s, 72°C 60 s) for 32 cycles, 72°C 10 min	298

### Statistics

The data are expressed as the mean ± SEM. The effects of CDT on the mean escape latency were analyzed by one-way ANOVA with a repeated-measure factor of sessions (number of days) followed by the least significant difference testing. The statistical significances of the other data were determined using ANOVA followed by least significant difference testing. Values of P < 0.05 were considered statistically significant.

## Results

### Effects of CDT on Aβ_1-42_ deposition in the hippocampus and cortex

**Figure 1:**
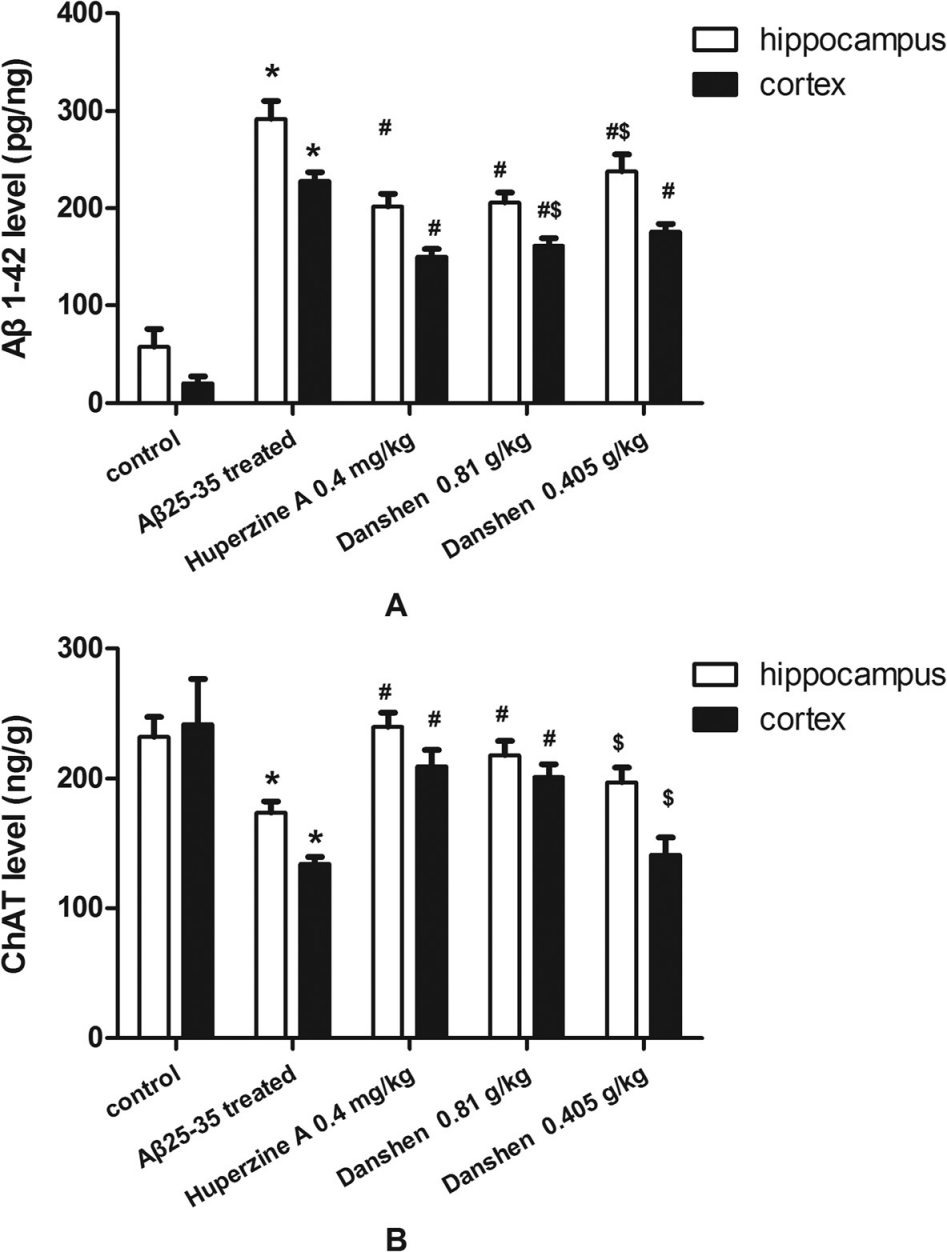
**Effects of CDT on Aβ**_**1-42 **_**deposition and ChAT Level in hippocampus and cortex. (A)** Aβ_1-42_ level in hippocampus and cortex of mice measured using ELISA; **(B)** ChAT level in hippocampus and cortex of mice measured using ELISA. Data are expressed the mean ± s.e.m for five samples per treatment group. ^*^p < 0.05 compared with control group; ^#^*p* < 0.05 compared with Aβ_25-35_ treated group; ^$^*p* < 0.05 compared with 0.4 mg/kg Huperzine A group.

To confirm the effect of CDT on Aβ_1-42_ deposition in Aβ_25-35_ induced AD model in mice, we first examined the Aβ_1-42_ deposition in hippocampus and cortex of mice by enzyme-linked immuno sorbent assay (ELISA). As compared with that of control group, Aβ_25-35_ peptide administration induced the marked Aβ_1-42_ protein deposition in hippocampus and cortex (Figure 1A) of mice. When combined with Aβ_25-35_ peptide treatment, LCDT actually could significantly attenuate the Aβ_1-42_ deposition in both of hippocampus and cortex (Figure 1A) (P < 0.05). Moreover, there was no significant difference between LCDT and Huperzine A group (P > 0.05, respectively) (Figure 1A).

### Effects of CDT on Aβ_25-35_-induced spatial memory impairment

**Figure 2:**
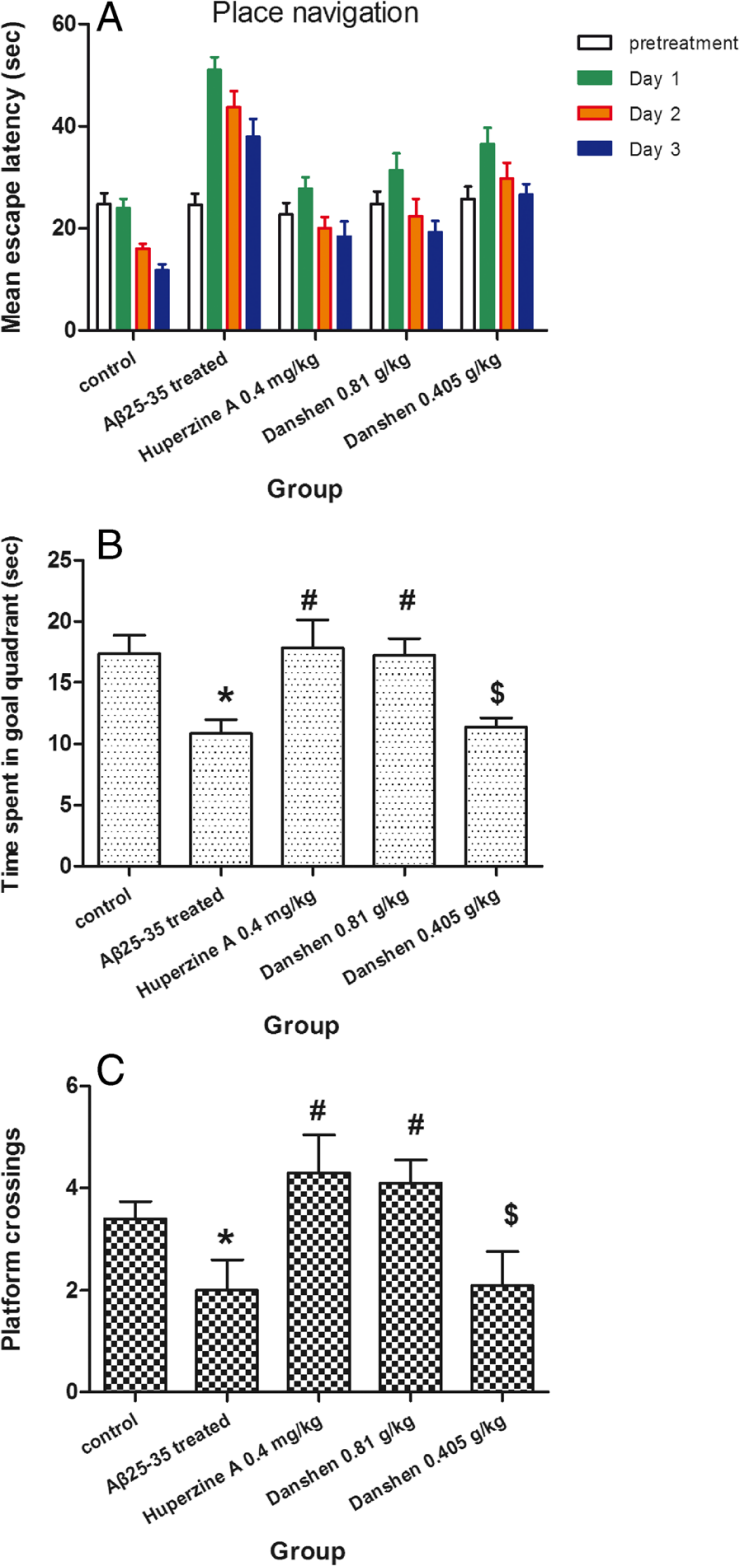
**Effects of CDT on Aβ**_**25-35**_**-induced spatial memory impairment. (A)** Effects of CDT on escape latency in the training trials of the water maze task; **(B)** Effects of CDT on the time spent in goal quadrant (Sec); **(C)** Effects of CDT on the number of platform crossings. Data are expressed the mean ± s.e.m for five samples per treatment group. ^*^p < 0.05 compared with control group; ^#^*p* < 0.05 compared with Aβ_25-35_ treated group; ^$^*p* < 0.05 compared with 0.4 mg/kg Huperzine A group.

ANOVA for repeated measures was conducted after Mauchly’s test of sphericity (F_(2)_ = 0.893, P = 0.082) in escape latency. There were significant differences in escape latency among the animals of the five groups (F_(2, 44)_ = 5.837, P = 0.006). The ANOVA for repeated measures followed by least significant difference testing revealed that Aβ_25-35_-induced model mice slowly arrived at the location of the platform, compared to the control group (P = 0.000). But either treatment with CDT at a dose of 0.81 or 0.405 g/kg or treatment with Huperzine A at a dose of 0.4 mg/kg significantly ameliorated these memory-impaired effects of Aβ_25-35_-induced mice on escape latencies as compared with the AD model group (P = 0.000, or P = 0.005, respectively). Moreover, there was no significant difference in escape latency between CDT and Huperzine A group (P > 0.05, respectively) (Figure 2A).

One day after the water maze test, we performed a probe trial to measure the maintenance of memory function. As shown in Figure 2B, the time spent in the target quadrant was significantly shorter in the Aβ_25-35_ treated group than in control group. The mice in the LCDT and Huperzine A group spent more time in the target quadrant than Aβ_25-35_-induced mice (P < 0.05). And treatment with LCDT and Huperzine A also markedly increased the number of platform crossings, which reduced notably in Aβ_25-35_-induced mice (P < 0.05) (Figure 2C).

### CDT protected mice from Aβ_25-35_-induced neuronal damage

**Figure 3:**
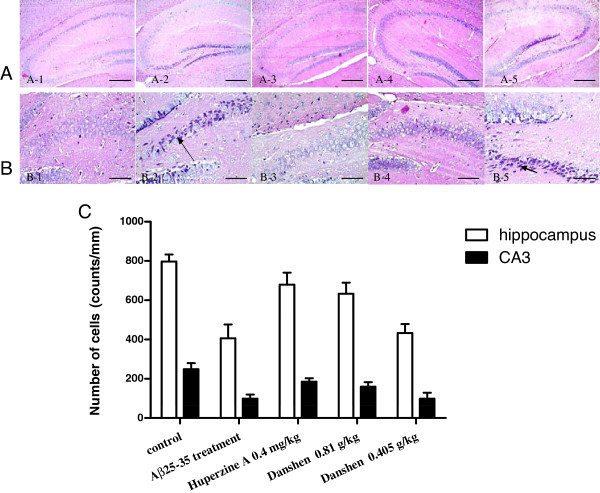
**Neuroprotection of CDT on Aβ**_**25-35**_**-induced neuronal damage. (A)** HE staining was used to evaluate the neuronal damage of hippocampus (Magnification × 200); **(B)** The damage of pyramidal cells in hippocampal CA3 region (Magnification × 800), the arrowheads represents the nuclear pyknosis. A large number of damaged neurons were seen in the CA3 of the hippocampus in Aβ_25-35_ treated mice (B-2). Compared with Aβ_25-35_ treated mice, there was less neuronal damage in the CA3 of the hippocampus of Huperzine A and LCDT treated mice; **(C)** Counts of the total number of cells in the hippocampus and CA3 (n = 18). The columns represent the means and SD of cell numbers in 24 animals. Data are expressed the mean ± s.e.m for five samples per treatment group. ^*^p < 0.05 compared with control group; ^#^*p* < 0.05 compared with Aβ_25-35_ treated group; ^$^*p* < 0.05 compared with 0.4 mg/kg Huperzine A group.

The histological photographs for the examination of neuronal damage from the differently treated mice groups are shown in Figure 3A and B. Aβ_25-35_ injection significantly reduced the number of neurons, resulted in a severe neuronal degeneration at hippocampus, especially in CA3, with much more nuclear pyknosis, nucleolus disappears and triangulated neuronal body than that of sham group. The Aβ_25-35_ treatment group had significantly lower cell counts in the hippocampus and in the areas CA3 when compared to the control group (Figure 3C). The extent of Aβ_25-35_-induced neuronal damage was significantly decreased at the hippocampus derived from the mice treated with LCDT. The groups treated with LCDT demonstrated a beneficial effect on the total cell number in the hippocampus than the Aβ_25-35_ treatment group (Figure 3C). The hippocampal subregion cell counts confirmed the effects of LCDT in CA3 (Figure 3C). Similarly, a much lower level of hippocampal neuron damage was observed in the mice treated with Huperzine A.

### Effects of CDT on ChAT Level in hippocampus and cortex

ELISA results also showed that Aβ_25-35_ peptide administration dramatically decreased ChAT protein level (Figure 1C and D, P < 0.05). ChAT protein level was significantly increased in the Huperzine A and LCDT groups in both of hippocampus and cortex (Figure 1B) compared to the Aβ_25-35_-induced mice (P < 0.05). Moreover, there were no differences between the LCDT and Huperzine A with regard to ChAT protein level not only in hippocampus but also in cortex (Figure 1).

### Effects of CDT on IL-6 and TNF-α levels in hippocampus

**Figure 4:**
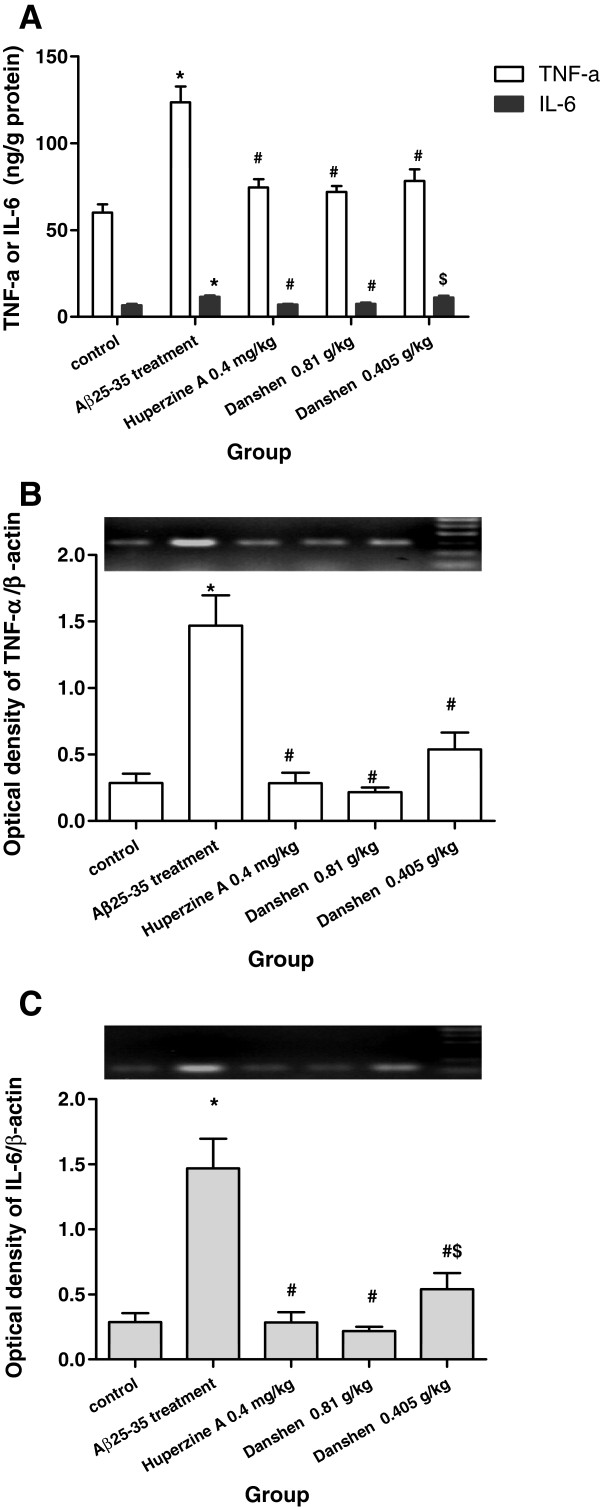
**Effects of CDT on TNF-α and IL-6 Levels in hippocampus of Aβ**_**25-35**_**-induced mice. (A-B)** The protein levels of TNF-α and IL-6 in hippocampus; **(C-D)** The mRNA levels of TNF-α and IL-6 in hippocampus. Data are expressed the mean ± s.e.m for five samples per treatment group. Data are expressed the mean ± s.e.m for five samples per treatment group. ^*^p < 0.05 compared with control group; ^#^*p* < 0.05 compared with Aβ_25-35_ treated group; ^$^*p* < 0.05 compared with 0.4 mg/kg Huperzine A group.

To confirm the anti-inflammatory activity of CDT, the mRNA and protein levels of IL-6 and TNF-α in hippocampus were measured using RT-PCR and ELISA, respectively. Aβ_25-35_ triggered a significant increase in all cytokines secretion levels (Figure 4) in hippocampus compared with those observed in control group. Administration of LCDT resulted in a significant reduction of IL-6 and TNF-α level in hippocampus as compared to Aβ_25-35_ -treated mice (P < 0.05). A similar effect was found in Huperzine A group. But treatment with SCDT just affected the TNF-α level in hippocampus.

Similarly, the mRNA level of IL-6 and TNF-α were also markedly stimulated by Aβ_25-35_ administration (Figure 4B,C) (P < 0.05). LCDT and SCDT treatment reversed the Aβ_25-35_-induced IL-6 and TNF-α mRNA over-expression (P < 0.05), and Huperzine A conferred a similar effect as compared with LCDT and more profound effect than SCDT on the mRNA expression of IL-6 (P < 0.05). Above results clearly demonstrated that CDT could effectively suppress IL-6 and TNF-α expression that was stimulated by Aβ_25-35_.

### Effects of CDT on BDNF and RACK1 Levels in hippocampus

**Figure 5:**
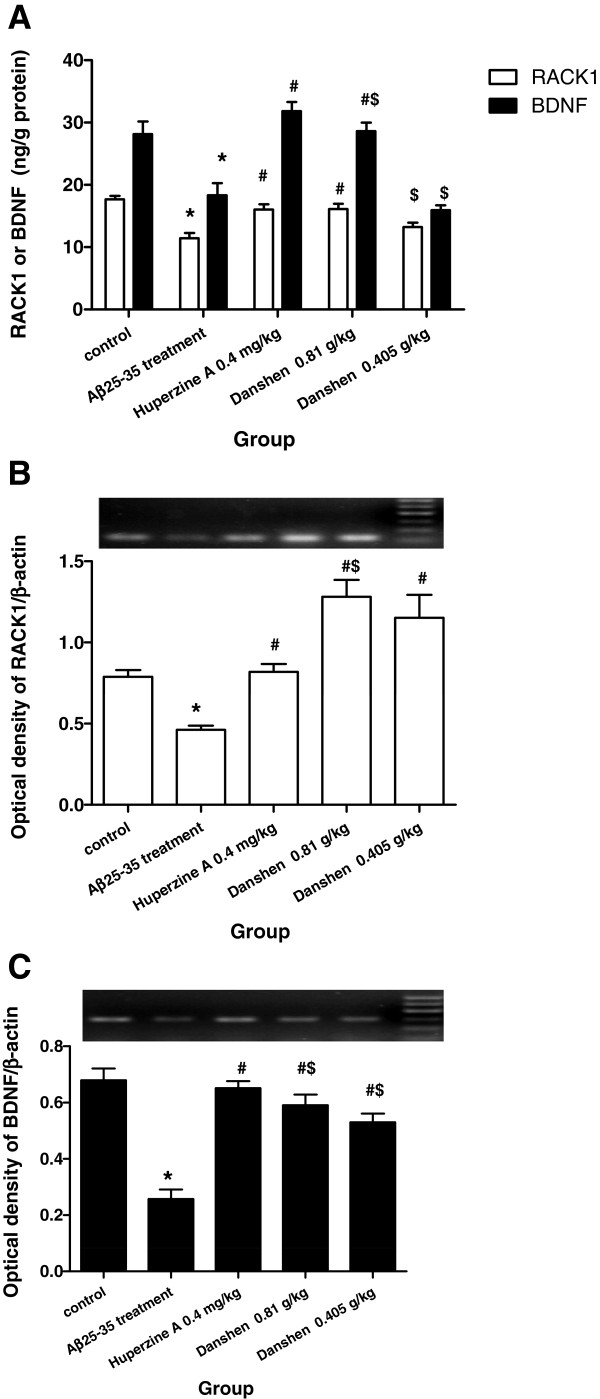
**Effect of CDT on RACK1 and BDNF Levels in hippocampus of Aβ**_**25-35**_**-induced mice. (A-B)** The protein levels of RACK1 and BDNF in hippocampus; **(C-D)** The mRNA levels of BDNF and RACK1 in hippocampus. Data are expressed the mean ± s.e.m for five samples per treatment group. Data are expressed the mean ± s.e.m for five samples per treatment group. ^*^p < 0.05 compared with control group; ^#^*p* < 0.05 compared with Aβ_25-35_ treated group; ^$^*p* < 0.05 compared with 0.4 mg/kg Huperzine A group.

To determine whether CDT influenced neurotrophins secretion in hippocampus, we also analyzed the levels of BDNF and RACK1 in hippocampus by ELISA. Aβ_25-35_ triggered a significant decrease in BDNF and RACK1 levels in hippocampus compared with those observed in control group (Figure 5A). LCDT almost restored BDNF and RACK1 levels to normal (Figure 5A) (P < 0.05). A similar effect was found in Huperzine A group.

To further bear out the neuroprotective effect of CDT, the mRNA level of BDNF and RACK1 was assayed by RT-PCR. In agreement with our ELISA data, BDNF mRNA level was decreased in Aβ_25-35_-induced mice too (Figure 5C) (P < 0.05). We found the notable enhancement of BDNF mRNA level no matter in CDT or Huperzine A treated mice (Figure 5C) (P < 0.05), but Huperzine A conferred more profound effects (P < 0.05), as compared with the other groups.

In addition to BDNF, RACK1 mRNA level was lessened in Aβ_25-35_-induced mice, as shown in Figure 5B (P < 0.05). Likewise, treatment with CDT and Huperzine A markedly strengthened the RACK1 mRNA expression in hippocampus when compared with Aβ_25-35_-induced mice (P < 0.05). Moreover, LCDT is more efficient in enhancing RACK1 mRNA expression than Huperzine A (P < 0.05).

## Discussion

The major finding of the present study was that pretreatment with the CDT prevented the spatial memory impairment and neuronal damage induced by i.c.v. injection of Aβ_25-35_. The mechanism of these neuroprotective effects may relate to elevated ChAT, RACK1 levels, and restored the balance between cytokines (IL-6 and TNF-α) and neurotrophins (BDNF) in the brain.

In Alzheimer’s disease, amyloid β (Aβ) protein is the main composite of senile plaques. Intracerebroventricular infusion of the active fragment of Aβ protein, Aβ_25-35_, has been shown to induce spatial learning and memory deficits in AD animal models [20,21]. Our results are consistent with previous reports that i.c.v. injection of Aβ_25-35_ induced significant spatial memory impairment measured by Morris water maze test [22]. In this study, we found that the escape latency was longer, and both of the time spent in the target quadrant and the numbers of platform crossings were shorter in the model group than those in the control group. Treatment with LCDT not only decreased the mean escape latency but also increased the time spent in the target quadrant and the numbers of platform crossings. Thus, it was reasonable to believe that LCDT could ameliorate the spatial memory impairment in Aβ_25-35_ treated mice.

The central cholinergic system plays an important role in learning and memory processes [23]. Loss of cholinergic neurons due to the neurotoxicity of Aβ, especially in hippocampus, is the major neuropathological feature that is associated with memory loss in AD [24,25]. Currently, the main clinical strategy is to increase ACh levels, modulate glial activation, cerebral blood flow, the amyloid cascade, and tau phosphorylation in brains with AD disease by acetylcholinesterase inhibitors [26]. However, choline acetyltransferase (ChAT) is one of the specific cholinergic marker proteins for monitoring the functional state of cholinergic neurons in the central nervous systems is ChAT, the biosynthetic enzyme for Ach [27]. Activation of ChAT could ultimately lead to synthesis sufficient Ach, may serve as a strategy for the treatment of memory impairment. In consideration of the main composition of CDT, *Savia miltiorrhiza*, is the novel acetylcholinesterase inhibitors [15]. The current work displayed that LCDT not only has a notable neuroprotective effect in Aβ_25-35_ treated mice, especially in CA3 of hippocampus and cortex via inhibiting the neuron damages but also significantly increased the ChAT protein level in hippocampus and cortex of Aβ_25-35_ induced mice. Thus, these results indicate that LCDT could be an effective approach for attenuating the neurotoxicity induced by Aβ_25-35_ via modulation of ChAT protein.

Neuroinflammatory responses of the central nervous system (CNS) are well-known features of Alzheimer’s disease (AD). Aβ not directly cause neuronal cell death but activate microglial cells to produce inflammatory factors. In turn, proinflammatory cytokines such as TNF-α and IL-6 had also been shown as an amplifier in the amyloid cascade process [28,29]. Overproduction of IL-6 and TNF-α were related to memory impairment [30,31], which were considered a histopathological hallmark of various neurological diseases in the brain [32]. Since hippocampus is more susceptible to these cytokine-induced inflammations [33], we analyzed the mRNA and protein levels of IL-6 and TNF-α in hippocampus and found that CDT (0.81 g/kg) administration prominently inhibited the production of pro-inflammatory mediators such as IL-6 and TNF-α. Thus, we suggest LCDT as a potential neuroprotective agent to prevent and treat neuroinflammation.

Recently, increasing evidence has showed that neuroinflammation may trigger neuroprotection or neurodegeneration through the neurotrophic system, such as BDNF in acute and chronic neurodegenerative. In AD, over-stimulated microglia but suppressed astrocyte functions resulted in the decrease of BDNF [34]. So imbalance of cytokines and neurotrophins has been put forward as the new mechanism of AD [35]. On the other hand, receptors for activated C kinase1 (RACK1), a family of proteins involved in anchoring activated PKCs to relevant subcellular compartments, is also deficient in the brain of alzheimer’s disease patients [36]. Presently study indicated that nuclear RACK1 localizes at the promoter IV region of the BDNF gene to regulate the expression of the BDNF gene [37]. To certain the effect of CDT on RACK1/BDNF signal pathway in AD mice, we further studied the mRNA and protein expression of RACK1 and BDNF in hippocampus of Aβ_25-35_-induced mice. Evidently, contrary to the anti-inflammatory effects, RACK1 and BDNF were shown to be lowered by Aβ_25-35_ treatment compared to the levels observed in sham mice. CDT treatment (0.81 g/kg) greatly rescued the Aβ_25-35_-induced RACK1/BDNF signal pathway in hippocampus. Thus, LCDT-mediated the modulation of RACK1/BDNF in hippocampus might contribute to neuroprotective effects of LCDT. Simutaneously, in present study, we found 0.81 g/kg CDT treatment significantly rescued the imbalance of cytokines (IL-6 and TNF-α) and neurotrophins (RACK1 and BDNF). We think this is due to over-expression cytokines (IL-6 and TNF-α) over activated microglia and resulted in the decrease of RACK1/BDNF signal pathway. Therefore, CDT restoration of RACK1/BDNF levels by inhibiting the cytokines levels.

## Conclusion

Here, taken together, the findings in the present study provided important information on the improvement effect of CDT on learning and memory in Aβ_25-35_-induced mice. Although CDT is well-known for the acetylcholinesterase inhibitors, it possesses anti-inflammatory activities and neurotrophins effects on Aβ_25-35_-induced mice independent of acetylcholinesterase inhibition.

## Competing interests

The authors declare that they have no competing interests.

## Authors’ contributions

LHW conceived and designed the study. YT, MQZ, WW and SKM performed many of the experiments and data analysis. LTL drafted the manuscript. LMZ was involved in the conception and design of the study and the supervision of experiments. All authors read the manuscript, contributed to its correction, and approved the final version.

## Pre-publication history

The pre-publication history for this paper can be accessed here:

http://www.biomedcentral.com/1472-6882/14/23/prepub
